# Improved NB Model Analysis of Earthquake Recurrence Interval Coefficient of Variation for Major Active Faults in the Hetao Graben and Northern Marginal Region

**DOI:** 10.3390/e28010107

**Published:** 2026-01-16

**Authors:** Jinchen Li, Xing Guo

**Affiliations:** 1 Institute of Geophysics, China Earthquake Administration, Beijing 100081, China; lijinchen1979@163.com; 2 Nuclear and Radiation Safety Center, Ministry of Ecology and Environment, Beijing 100082, China

**Keywords:** coefficient of variation, paleoseismology, Brownian Passage Time distribution, seismic hazard assessment

## Abstract

This study presents an improved Nishenko–Buland (NB) model to address systematic biases in estimating the coefficient of variation for earthquake recurrence intervals based on a normalizing function $\frac{T}{T_{ave}}$. Through Monte Carlo simulations, we demonstrate that traditional NB methods significantly underestimate the coefficient of variation when applied to limited paleoseismic datasets, with deviations reaching between 30 and 40% for small sample sizes. We developed a linear transformation and iterative optimization approach that corrects these statistical biases by standardizing recurrence interval data from different sample sizes to conform to a common standardized distribution. Application to 26 fault segments across 15 major active faults in the Hetao graben system yields a corrected coefficient of variation of α = 0.381, representing a 24% increase over the traditional method (α_0_ = 0.307). This correction demonstrates that conventional approaches systematically underestimate earthquake recurrence variability, potentially compromising seismic hazard assessments. The improved model successfully eliminates sampling bias through iterative convergence, providing more reliable parameters for probability distributions in renewal-based earthquake forecasting.

## 1. Introduction

Based on the elastic rebound theory [1], after a major earthquake occurs on a fault, sufficient energy must accumulate before the next major earthquake can occur. To describe this quasi-periodicity, Utsu, Rikitake, and Hagiwara proposed a renewal model [2,3,4]. Based on paleoseismic and historical earthquake data, researchers have proposed various probability distributions for the renewal model, including the biexponential distribution [2], the Gaussian distribution [3], the lognormal distribution [5], the Weibull distribution and gamma distribution [6], and the Brownian Passage Time (BPT) distribution [7,8].

For an active fault, if sufficient recurrence interval data are available, the probability distribution of major earthquake recurrence can be directly determined through statistical analysis. However, due to the extended recurrence intervals of earthquakes, most active faults only have limited recurrence interval data, which are insufficient for establishing reliable distributions or estimating distribution parameters.

When recurrence interval data for an individual fault are inadequate, assuming that multiple faults within a region of similar seismic background exhibit consistent variability and analogous distribution patterns becomes a practical alternative. Nishenko and Buland introduced a normalizing function $\frac{T}{T_{ave}}$, where $T_{ave}$ represents the observed average recurrence interval for a specific fault or fault segment, and T denotes an individual recurrence interval [5]. Utilizing this normalizing function $\frac{T}{T_{ave}}$ enables the integration of recurrence interval data from multiple distinct faults, allowing for the collective statistical analysis to derive a generic distribution pattern or coefficient of variation.

In recent years, the Nishenko–Buland (NB) model has continued to be widely applied in earthquake prediction and seismic hazard analysis as a statistical tool for determining the probability distribution of major earthquake recurrence intervals. The application of the NB model enables relatively accurate assessment of recurrence time intervals for high-magnitude earthquakes [9,10,11,12,13].

However, the NB model presents a significant limitation: the mean value $T_{ave}$ of limited recurrence intervals on a fault does not equal the true average recurrence interval, potentially introducing substantial bias in results derived from $\frac{T}{T_{ave}}$ statistics [14]. Furthermore, different sample sizes of recurrence intervals yield varying degrees of deviation between the $\frac{T}{T_{ave}}$ derived distribution and the actual distribution.

To conduct a statistical analysis of this bias, we employed the commonly used Brownian Passage Time (BPT) distribution as an example to quantify the deviation in the coefficient of variation between $\frac{T}{T_{ave}}$-derived distributions under different interval numbers and the actual distributions.

Subsequently, based on this bias analysis, we propose an improved NB model that employs linear transformation to correct the deviation between small-sample statistics and actual distributions. This approach ensures that data from different recurrence interval sample sizes conform to the same distribution, thereby yielding an unbiased generic coefficient of variation.

Finally, this study focuses on 15 major faults along the northern margin of the Hetao graben as research subjects. Assuming that the major earthquake recurrence of these 15 faults all follow a BPT distribution with a uniform coefficient of variation, we apply the proposed improved NB model to derive a generic coefficient of variation value through statistical analysis.

## 2. Theory and Methods for Improving the NB Model

### 2.1. Problems in the Traditional NB Model

Nishenko and Buland introduced a normalizing function $\frac{T}{T_{ave}}$ to integrate recurrence interval data from multiple faults, enabling the statistical derivation of a generic coefficient of variation. The NB model addresses the issue of insufficient data on individual faults and provides a reliable probability distribution. However, the mean value ($T_{ave}$) of limited recurrence intervals on a fault does not equal the true mean recurrence interval, which may lead to deviations in both the distribution pattern and coefficient of variation α of $\frac{T}{T_{ave}}$ from the actual distribution [14]. Furthermore, the deviation may vary depending on the sample size of recurrence intervals.

To address this issue, we employ a Monte Carlo simulation to quantify and analyze the sampling bias inherent in coefficient of variation estimation.

Our Monte Carlo simulation employs a robust numerical framework to generate synthetic BPT-distributed recurrence intervals. To generate random variates conforming to the BPT distribution, we implement an inverse transform sampling method: (1) Generate a uniform random number U from the interval [0, 1]. (2) Numerically solve the equation U = F(T), where F(T) denotes the cumulative distribution function (CDF) of the BPT distribution. (3) The solution T represents a BPT-distributed random variate with parameters μ and α. (4) Each simulation scenario (characterized by specific α_0_ and sample size *n*) comprises 100,000 independent replications to ensure statistical stability, with convergence verified by calculating 95% confidence intervals for the estimated coefficient of variation.

Given an original BPT distribution with a predefined coefficient of variation α_0_, we generate multiple datasets through iterative random sampling, with each dataset comprising *n* recurrence interval samples (*n* > 1). We subsequently evaluate the systematic deviation between the empirically derived coefficient of variation α, calculated from the $\frac{T}{T_{ave}}$ statistics of the *n* samples, and the true coefficient of variation α_0_.

Based on paleoseismic and historical earthquake data, researchers have proposed various probability distributions for the renewal model. The BPT model offers a more explicit physical interpretation and is widely employed for recurrence probability assessments in probabilistic seismic hazard assessment [15]. Therefore, this study assumes that the recurrence probability distribution follows a BPT distribution.

The BPT model, introduced by Ellsworth et al. and Matthews et al., is grounded in Reid’s elastic rebound theory [7,8]. It assumes that tectonic stress accumulates steadily but is influenced by random fluctuations, that earthquakes occur once stress reaches a critical threshold, and that stress resets to a fixed lower limit following each event. Its probability density function is expressed as follows:

(1) $$f(T)=\sqrt{\frac{\mu }{2\pi \alpha^{2}T^{3}}}e^{-\frac{{\left(T-\mu \right)}^{2}}{2\mu \alpha^{2}T}}$$

where T represents the variable for recurrence intervals, μ is the average recurrence interval of large earthquakes on active faults, and α is the coefficient of variation in the recurrence interval. This coefficient of variation α reflects the variability of the recurrence intervals for large earthquakes and can be obtained via the statistical analysis of large amounts of recurrence data.

First, assuming conformity to the BPT distribution and given a coefficient of variation value α_0_, recurrence interval data can be randomly generated using the Monte Carlo method. Since the normalized $\frac{T}{T_{ave}}$ is dimensionless, it is only necessary to set the coefficient of variation α_0_ for the BPT distribution, while the μ value can be set arbitrarily. In each simulation, the randomly generated recurrence interval data is set to *n* (*n* > 1). Through numerous simulations (e.g., 100,000 iterations), the deviation between the coefficient of variation α obtained from $\frac{T}{T_{ave}}$ statistics and the actual coefficient of variation α_0_ can be determined when the recurrence interval sample size is *n*.

**Figure 1:**
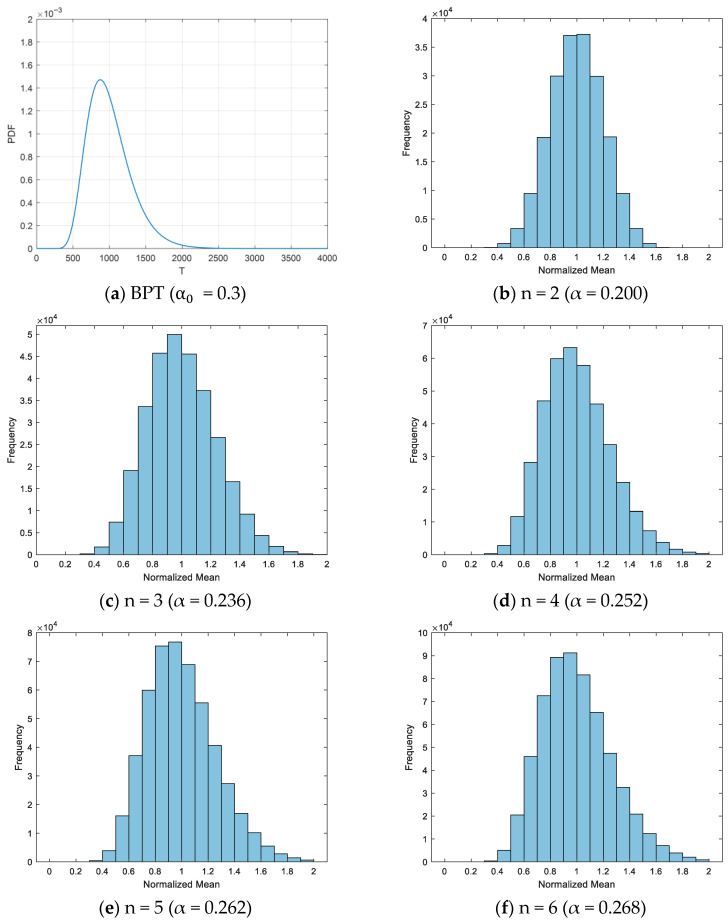
BPT Distribution vs. $\frac{T}{T_{ave}}$ Distribution Derived from Small Sample Data.

Figure 1 presents the results for an initial distribution with α_0_ = 0.3 and recurrence interval sample sizes *n* of 2, 3, 4, 5, and 6, yielding coefficient of variation values α of 0.200, 0.236, 0.252, 0.262, and 0.268, respectively, based on $\frac{T}{T_{ave}}$ statistics. The statistical results demonstrate that the coefficient of variation in the distribution obtained from small-sample $\frac{T}{T_{ave}}$ statistics exhibits significant deviation from the actual coefficient of variation, with smaller sample sizes producing larger deviations.

Second, considering that different coefficient of variation values may also contribute to the deviation between the coefficient of variation obtained from $\frac{T}{T_{ave}}$ statistics based on small samples and the actual coefficient of variation, this study further selected six different initial coefficients of variation (α_0_): 0.1, 0.2, 0.3, 0.4, 0.5, and 0.6. For each α_0_ value, 100,000 simulations were performed to investigate the deviation between the coefficient of variation (α) of the BPT distribution derived from $\frac{T}{T_{ave}}$ statistics based on different small sample sizes and the true values.

Table 1 presents the deviations between the BPT distribution coefficient of variation (α) obtained from $\frac{T}{T_{ave}}$ statistics and the true values (α_0_) under different initial coefficient of variation scenarios. The results in Table 1 demonstrate that the deviation between the coefficient of variation (α) of the BPT distribution derived from $\frac{T}{T_{ave}}$ statistics based on small samples and the actual value (α_0_) is influenced not only by the sample size but also by the value of the true coefficient of variation (α_0_). Specifically, smaller actual coefficients of variation (α_0_) correspond to smaller relative deviations of α from α_0_.

### 2.2. Improvement of the NB Model

According to the statistical results shown in Figure 1, the coefficient of variation in the $\frac{T}{T_{ave}}$ distribution obtained from small sample statistics exhibits substantial deviation from the actual variation, with this deviation becoming more pronounced as the sample size decreases.

When recurrence interval sample sizes differ across multiple faults, and the $\frac{T}{T_{ave}}$ from different sample sizes do not follow the same distribution, they cannot be statistically combined, as non-identical distributions violate the assumptions of joint statistical analysis. Consequently, the traditional NB method introduces a significant statistical bias.

To eliminate this bias, this study proposes a linear transformation approach that corrects each $\frac{T}{T_{ave}}$ data point to ensure they conform to the same distribution with identical means and coefficients of variation, thereby eliminating the statistical bias inherent in the traditional NB method.

To adjust a distribution with mean = 1 and coefficient of variation = α to a distribution with mean = 1 and coefficient of variation = α_0_, the following linear transformation can be applied.

Let X be the original variable and Y be the target variable, then the transformation formula is as follows:

Y = a × X + b(2)

where a = original coefficient of variation/new coefficient of variation = α_0_/α; b = new mean − a × original mean = 1 − a.

The linear transformation defined in Equation (2) is mathematically justified for the BPT distribution through the following theoretical framework. Similar to the traditional standardization method based on $\frac{T}{T_{ave}}$—where the $\frac{T}{T_{ave}}$ distribution does not strictly conform to the BPT distribution—our proposed standardization method employing linearly transformed $\frac{T}{T_{ave}}$ values also represents an approximation.

It is important to acknowledge that the BPT distribution lacks shape invariance under linear transformations (unlike the Gaussian distribution). Theoretically, applying Y = aX + b alters the skewness of the distribution, which could potentially affect probability calculations. However, our Monte Carlo validation demonstrates that this shape distortion has negligible impact on practical probability estimation for the following reasons: (1) the BPT distribution’s skewness is already modest in the typical range of coefficients of variation (α < 0.7) relevant to seismic applications; (2) our transformation preserves the two most statistically significant parameters—the mean recurrence interval and coefficient of variation—which dominate the tail probability calculations critical for hazard assessment.

Specifically, the linear transformation Y = aX + b preserves the essential distributional characteristics by maintaining these two fundamental parameters. This preservation of key statistical properties, combined with the minimal impact of shape distortion on probability calculations, renders the proposed approach both practically viable and methodologically sound.

For a random variable X with mean E[X] = 1 and coefficient of variation CV(X) = α, the linear transformation Y = aX + b yields the following:

E[Y] = aE[X] + b = a(1) + (1 − a) = 1(3)

Var(Y) = a^2^Var(X) = a^2^α^2^ (4)

(5) $$\mathrm{CV}(Y)=\sqrt{Var(Y)/E[Y]}=a\alpha /1=(\alpha_{0}/\alpha )\cdot \alpha =\alpha_{0}$$

This demonstrates that the transformation successfully standardizes the mean to 1 and the coefficient of variation to the target value α_0_.

Example:

In Figure 1, the initial distribution has a coefficient of variation of 0.300. With a sample size of 3, the Monte Carlo simulation yields a coefficient of variation of 0.236. Therefore, to adjust the $\frac{T}{T_{ave}}$ distribution to achieve a coefficient of variation of 0.300, each $\frac{T}{T_{ave}}$ value requires a linear transformation. Thus, a = 0.300/0.236 ≈ 1.271; b = 1 − 1.271 × 1 = −0.271. The transformation formula becomes Y = 1.271X − 0.271.

To eliminate the influence of variance heterogeneity between different groups, standardization processing is applied to each group of data, ensuring all variables possess identical means and coefficients of variation before conducting $\frac{T}{T_{ave}}$-based statistical analysis.

### 2.3. Iterative Optimization Method

As shown in Table 1, the deviation between the coefficient of variation α derived from the BPT distribution of $\frac{T}{T_{ave}}$ based on different small samples and the actual value α_0_ is not only related to the sample size, but also to the magnitude of the actual distribution’s coefficient of variation α_0_.

In other words, for different actual coefficients of variation, the ratio a between the new coefficient of variation and the original coefficient of variation varies. Since the actual coefficient of variation is unknown, we can only initially set a possible coefficient of variation and then continuously optimize the parameter a through an iterative approach to improve the statistical results based on small-sample $\frac{T}{T_{ave}}$.

Therefore, we propose an iterative optimization method with the following algorithmic procedure:

Step 1: Initialization Set the initial value of α to α_0_.

Through the traditional NB method, obtain an initial coefficient of variation α_0_ based on actual $\frac{T}{T_{ave}}$ statistics.

Step 2: Standardization transformation

Employ the Monte Carlo method to statistically determine the deviation ratio a between the new and original coefficients of variation under different sample conditions. Then apply Equation (2) to perform a linear transformation on all actual $\frac{T}{T_{ave}}$ data according to their respective sample sizes, yielding a new coefficient of variation α_1_.

Step 3: Parameter update

Use α_1_ as the new initial value of α and employ the Monte Carlo method to statistically determine the deviation ratio a between the new and original coefficients of variation under different sample conditions. Then apply Equation (2) to perform a linear transformation on all actual $\frac{T}{T_{ave}}$ data according to their respective sample sizes, obtaining a new coefficient of variation α_2_.

Step 4: Convergence criterion

Calculate |α_k+1_ − α_k_|. If |α_k+1_ − α_k_| > 0.0005, repeat Step 3. If |α_k+1_ − α_k_| ≤ 0.0005, terminate the iteration. The threshold of 0.0005 is chosen to ensure the coefficient of variation is accurate to three decimal places. Where αk represents the k-th iteration result, and α_k+1_ represents the (k + 1)-th iteration result. Through continuous iteration, an optimized coefficient of variation α can be obtained.

It is acknowledged that this constitutes an empirical iterative method rather than a theoretically guaranteed algorithm. Nevertheless, the stability and convergence of this method have been empirically observed in the present study’s simulations, supported by the following properties.

Statistical robustness: The 100,000-replication Monte Carlo simulations at each iteration yield standard errors < 0.001 for coefficient of variation estimates, preventing oscillatory behavior attributable to sampling noise.

Empirically observed error reduction: In our simulations, each iteration was consistently observed to reduce the systematic bias between the empirically observed distribution and the target distribution with the true (unknown) coefficient of variation α. While not mathematically proven, this monotonic convergence pattern was stable across all simulation scenarios examined in this study.

## 3. Paleoseismic Data of Major Faults Along the Northern Margin of the Hetao Graben and Application of the Improved NB Model

### 3.1. Fault Systems and Seismic Data in the Study Area

**Figure 2:**
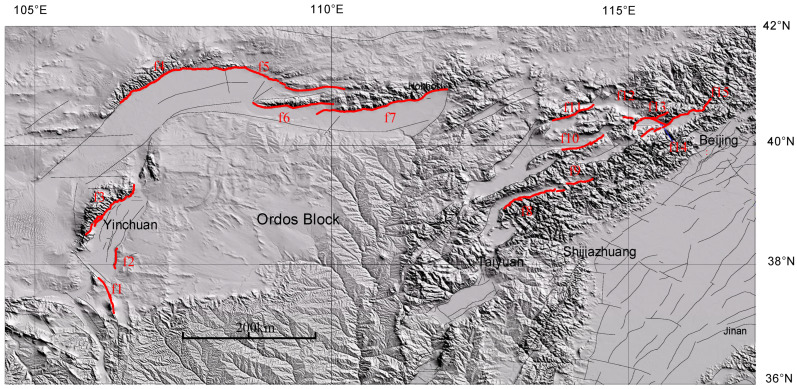
Active faults in the Hetao Graben and northern margin (f1: Luoshan eastern piedmont fault; f2: Yellow River–Lingwu fault; f3: Helanshan eastern piedmont fault; f4: Langshan piedmont fault; f5: Seertenshan piedmont fault; f6: Wulashan piedmont fault; f7: Daqingshan piedmont fault; f8: Wutaishan northern piedmont fault; f9: Taibai–Weishan northern piedmont fault; f10: Yangyuan Basin southern margin fault; f11: Yanggao–Tianzhen northern margin fault; f12: Xuanhua Basin southern margin fault; f13: Huai-Zhuo Basin northern margin fault; f14: Xinbaoan–Shacheng fault; f15: Yanfan Basin northern margin fault).

This study focuses on the Hetao Graben and its northern margin (105–117° E, 38.5–42° N; Figure 2). Situated along the northern edge of the Ordos Block, this region represents a transitional zone between the North China Craton and the Alxa Block and is characterized by comparable tectonic evolutionary histories and seismic activity patterns.

The regional active fault system primarily comprises NE–NEE trending structures, including the Yinchuan Graben and Hetao Graben, along with conjugate NW-trending faults [16,17]. The controlling boundary faults include the Langshan Piedmont Fault, Wulashan Piedmont Fault, Baotou Fault, Daqingshan Piedmont Fault, and the northern margin fault of the Yanfan Basin. These fault zones represent active tectonic structures since the Quaternary, exhibiting similar normal fault characteristics and extensional tectonic features.

Seismic activity in this region displays distinct zonal distribution patterns, primarily concentrated along active fault zones. The area has experienced significant historical earthquakes, including the 1739 M 8.0 Pingluo earthquake and the 1888 M 6.0 Wulate earthquake. Currently, the region maintains relatively high levels of small-to-moderate seismic activity [18]. Focal mechanism solutions predominantly show normal faulting, reflecting consistent regional stress field conditions.

Based on the similar geological and structural background, unified geodynamic environment, and consistent seismic activity patterns, earthquake data from different fault zones within this region can be integrated for comprehensive statistical analysis. This provides a reliable database for investigating the overall seismic activity patterns of the Hetao Graben system.

Fifteen faults within the Hetao rift zone and its northern marginal region contain relatively comprehensive paleoseismic records. However, the completeness of paleoseismic data varies significantly among different fault segments. Therefore, establishing robust paleoseismic data screening criteria is essential for reliable assessment of major earthquake recurrence probabilities.

The paleoseismic data screening protocol employed in this study adheres to the following criteria: (1) Priority is given to the most recently published authoritative paleoseismic investigations; (2) Events lacking either upper or lower age constraints are excluded from recurrence interval calculations due to excessive temporal uncertainty; (3) Only seismic events consistent with quasi-periodic characteristic earthquake models are considered; (4) Paleoseismic sequences on faults with relatively small upper magnitude limits (<M7.0) are not considered, as such faults are unlikely to produce surface ruptures; (5) Paleoseismic events occurring during intervals characterized by evident record gaps or disputed paleoseismic completeness are excluded from the analysis.

### 3.2. Application of the Improved NB Model and Calculation of the Coefficient of Variation

**Figure 3:**
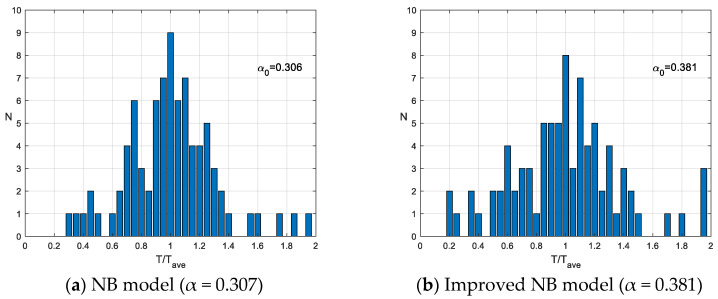
$\frac{T}{T_{ave}}$ distributions based on traditional and improved NB models.

Based on paleoseismic data from 26 fault segments across 15 major fault zones within the Hetao Graben and its northern marginal region (Table 2), statistical analysis of $\frac{T}{T_{ave}}$ using the NB method yields a general coefficient of variation α_0_ = 0.307 (Figure 3a). The paleoseismic dating data in Table 2 exhibit substantial uncertainties. The NB model statistics employed in this study do not account for such uncertainties; instead, they directly adopt the midpoint values of the dating ranges. This dating uncertainty falls within the category of epistemic uncertainty. To properly propagate paleoseismic dating uncertainties in subsequent recurrence probability calculations, quantitative approaches such as logic tree analysis or Bayesian inference could be implemented to provide a more comprehensive assessment of the reliability of probabilistic forecasts.

Subsequently, following the improved BPT model proposed in this study, iterative optimization was performed on the statistical results. The iterative process is shown in Table 3, where convergence to α = 0.381 was achieved after four iterations (Figure 3b). The final statistical distribution is presented in Figure 1b, with the horizontal axis representing the linearly transformed $\frac{T}{T_{ave}}$ values. The results demonstrate that the improved NB model proposed in this study, incorporating linear transformation of $\frac{T}{T_{ave}}$ and iterative optimization, yields a coefficient of variation α = 0.381, which is substantially larger than the coefficient of variation α_0_ = 0.307 obtained through the conventional NB method. In the traditional NB approach, the statistical analysis of $\frac{T}{T_{ave}}$ based on small samples exhibits significant bias in estimating the coefficient of variation in actual recurrence intervals, leading to substantial underestimation of the coefficient of variation.

**Figure 4:**
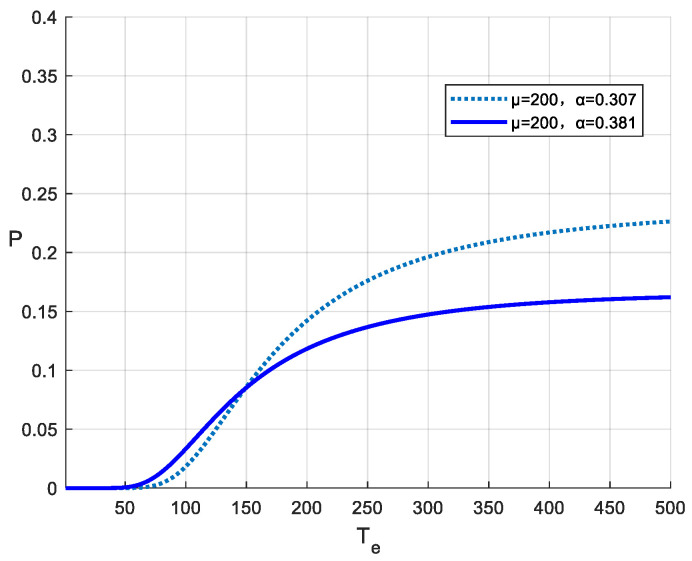
Comparison of earthquake occurrence probabilities over the next 10 years between the conventional BPT and improved BPT models. T_e_ represents the elapsed time since the last major earthquake.

To examine the influence of different methods for estimating the coefficient of variation (α) on earthquake occurrence probability calculations, this study presents a simple computational example. Earthquake recurrence probabilities were calculated for a future time period using the BPT distribution with μ = 200 years and ΔT = 10 years, employing α = 0.307 and α = 0.381, respectively. The results reveal that using 0.75μ as a threshold, the probability curve with α = 0.381 yields higher values before this point, whereas the curve with α = 0.307 produces higher probabilities beyond it (Figure 4). This demonstrates that substantial differences exist in earthquake occurrence probability estimates between the improved NB model proposed in this study and the conventional NB model. Furthermore, sensitivity analyses indicate that these differences vary with different combinations of μ and ΔT, with the crossover point typically occurring within the range of 0.7–0.8μ.

## 4. Discussions

### 4.1. Statistical Bias Correction and Model Performance

The improved NB model successfully addresses the systematic bias introduced by small sample sizes. Our Monte Carlo simulations demonstrate that the coefficient of variation derived from $\frac{T}{T_{ave}}$ statistics exhibits substantial deviation from true values, with deviations ranging from 30 to 40% for samples of 2–3 intervals. This bias correction is particularly significant given that most active faults worldwide possess limited paleoseismic records [8,25]. The iterative optimization approach ensures convergence to an unbiased coefficient of variation (α = 0.381 versus α_0_ = 0.307 for the traditional NB method), representing a 24% increase that reflects the true variability of earthquake recurrence intervals.

### 4.2. Methodological Limitations

The proposed methodology is theoretically applicable to any fault system where the fundamental assumptions of the renewal model hold, including the quasi-periodic behavior and stress accumulation-release cycles [1]. However, several limitations must be acknowledged. Not all fault systems exhibit the characteristic earthquake behavior required for renewal models, as some demonstrate complex, non-periodic rupture patterns influenced by fault interactions, triggered seismicity, or variable stress loading rates [26]. Additionally, the assumption of BPT distribution uniformity across different fault segments may oversimplify the complexity of earthquake systems, where factors such as fault geometry, stress heterogeneity, and multi-fault interactions can introduce significant variations in recurrence patterns. Consequently, applying a uniform distribution model may fail to reflect fault-to-fault heterogeneity, potentially leading to systematic biases in seismic hazard assessment. Finally, the method’s reliability remains contingent upon the quality and completeness of paleoseismic records, which are often incomplete due to preservation bias and limited temporal coverage.

### 4.3. Scientific Significance and Implications for Seismic Hazard Assessment

This study represents a significant methodological advancement in earthquake probability modeling by providing a statistically robust framework for coefficient of variation estimation in data-limited environments. The corrected coefficient of variation (α = 0.381) for the Hetao Graben system aligns well with global estimates for continental normal fault systems, which typically range from 0.3 to 0.5 [7]. The improved NB model enhances the reliability of probabilistic seismic hazard assessments by eliminating systematic biases that previously led to conservative risk estimates. This advancement is particularly valuable for regions with limited instrumental seismic records but rich paleoseismic histories, such as continental intraplate settings.

### 4.4. Future Research Directions and Model Development

Future research should focus on expanding the database of high-quality paleoseismic records to enable more refined statistical analyses across diverse tectonic settings and fault characteristics. Investigation of coefficient of variation dependencies on fault-specific parameters such as slip rate, maximum magnitude, fault length, and structural complexity would enhance model sophistication [27,28]. The development of hierarchical Bayesian approaches could better accommodate uncertainties in paleoseismic age determinations and provide more robust parameter estimation frameworks. Additionally, incorporating machine learning techniques and big data analytics may reveal previously unrecognized patterns in earthquake recurrence variability. Extension of the methodology to other probability distributions beyond BPT, including mixed distributions that account for both characteristic and non-characteristic earthquake behavior, represents another promising avenue for methodological advancement.

## 5. Conclusions

This study presents a significant methodological advancement in earthquake probability modeling through the development of an improved Nishenko–Buland (NB) model that addresses critical statistical biases inherent in traditional approaches. Our research successfully identifies and corrects systematic underestimation of coefficient of variation values when applying the conventional NB method to limited paleoseismic datasets. Through extensive Monte Carlo simulations, we demonstrated that small sample sizes introduce substantial deviations between observed and true coefficients of variation, with deviations ranging from 30 to 40% for datasets containing only 2–3 recurrence intervals. The magnitude of this bias is not only dependent on sample size but also correlates with the true coefficient of variation value, necessitating the iterative optimization approach we developed.

The application of our improved NB model to 26 fault segments across 15 major active faults in the Hetao Graben system yields a corrected coefficient of variation of α = 0.381, representing a 24% increase over the traditional NB method result (α_0_ = 0.307). This correction demonstrates that conventional approaches systematically underestimate earthquake recurrence variability, potentially leading to overconfident probability estimates in seismic hazard assessments. The linear transformation and iterative optimization procedures ensure that recurrence interval data from different sample sizes conform to the same statistical distribution, eliminating the bias that previously compromised joint statistical analyses. Our methodology successfully converges within four iterations, providing a robust and computationally efficient solution for coefficient of variation estimation in data-limited environments.

The corrected coefficient of variation value (α = 0.381) for the Hetao Graben system aligns well with global estimates for continental normal fault systems and provides critical input parameters for probabilistic seismic hazard assessment in this seismically active region. This finding has profound implications for earthquake risk evaluation and seismic safety considerations, as accurate variability estimates are essential for reliable probability calculations and appropriate risk mitigation strategies. The improved model addresses a fundamental limitation in renewal-based earthquake forecasting by providing unbiased statistical parameters that better reflect the true quasi-periodic nature of characteristic earthquakes along active fault systems.

The broader scientific significance of this research extends beyond regional applications to provide a standardized framework for coefficient of variation determination in any tectonic setting where sufficient paleoseismic data exist. The methodology is particularly valuable for continental intraplate regions and other seismically active areas with limited instrumental records but rich paleoseismic histories. Future applications of this improved NB model will enhance the reliability of long-term earthquake probability assessments worldwide, contributing to more accurate seismic hazard models and ultimately supporting evidence-based seismic risk management strategies. The theoretical framework developed here establishes a foundation for further refinements in earthquake recurrence modeling and represents a crucial step toward eliminating statistical biases that have long affected probabilistic seismic hazard assessment methodologies.

## Figures and Tables

**Table 1:** Relationship between the coefficient of variation α of the BPT distribution derived from $\frac{T}{T_{ave}}$ statistics and the initial coefficient of variation α_0_.

Sample Size of Intervals	α_0_ = 0.1	α_0_ = 0.2	α_0_ = 0.3	α_0_ = 0.4	α_0_ = 0.5	α_0_ = 0.6
2	α = 0.702α_0_	α = 0.689α_0_	α = 0.665α_0_	α = 0.637α_0_	α = 0.610α_0_	α = 0.582α_0_
3	α = 0.814α_0_	α = 0.802α_0_	α = 0.783α_0_	α = 0.763α_0_	α = 0.734α_0_	α = 0.707α_0_
4	α = 0.862α_0_	α = 0.854α_0_	α = 0.837α_0_	α = 0.820α_0_	α = 0.798α_0_	α = 0.775α_0_
5	α = 0.894α_0_	α = 0.884α_0_	α = 0.871α_0_	α = 0.855α_0_	α = 0.837α_0_	α = 0.816α_0_
6	α = 0.911α_0_	α = 0.904α_0_	α = 0.896α_0_	α = 0.880α_0_	α = 0.864α_0_	α = 0.846α_0_

**Table 2:** Paleoseismic data for 15 active faults in the Hetao Graben and northern margin.

No.	Fault	Segment	Paleoseismic Events	Number of Intervals	$\frac{T}{T_{ave}}$	Sources
f1	Luoshan eastern piedmont fault		E1: 8200 ± 600 E2: 5020 ± 70 E3: 3331 ± 92 E4: 464	3	1.2326 0.6547 1.1128	[19]
f2	Yellow River–Lingwu fault	Lingwu segment	E1: 27,150 ± 778 E2: 20,000 E3: 13,070 ± 60 E4: 10,586 ± 50 E5: 6000	4	1.3522 1.3106 0.4698 0.8673	[20]
f3	Helanshan eastern piedmont fault		E1: 8240 ± 170 E2: 6330 ± 80 E3: 4760 ± 80 E4: 2675 ± 70 E5: 286	4	0.9605 0.7895 1.0485 1.2014	[19]
f4	Langshan piedmont fault	Eastern Xibulong segment	E1: 3990 E2: 3655 E3: 2990 E4: 2380	3	0.6242 1.2391 1.1366	[21]
f5	Seertenshan piedmont fault	Dashetai segment	E1: 31,690 ± 1770 E2: 23,000 ± 1320 E3: 15,420 ± 870 E4: 7440 ± 440	3	1.0751 0.9377 0.9872	[21]
		Wulanhudong segment	E1: 25,130 ± 1430 E2: 14,570 ± 820 E3: 11,660 ± 650 E4: 7220 ± 400	3	1.7688 0.4874 0.7437	
f6	Wulashan piedmont fault	Gongmiaozi–Heshunzhuang segment	E1: 7215 ± 255 E2: 5935 ± 45 E3: 3645 ± 55 E4: 1655 ± 185	3	0.6919 1.2378 1.0703	[21]
		Heshunzhuang–Baotou segment	E1: 23,860 ± 1000 E2: 17,425 ± 970 E3: 16,500 ± 600 E4: 13,400 ± 600 E5: 11,850 ± 830 E6: 8385 ± 470 E7: 4130 ± 78	6	1.9569 0.2813 0.9427 0.4714 1.0537 1.2940	
f7	Daqingshan piedmont fault	Tumd Right Banner segment	E1: 10,309 ± 991 E2: 8760 ± 500 E3: 4545 ± 466 E4: 3650 ± 280 E5: 1176	4	0.6784 1.8461 0.3920 1.0835	[22]
		Tumd Left Banner segment	E1: 11,000 E2: 9000 E3: 7000 E4: 4000 E5: 2000	4	0.8889 0.8889 1.3333 0.8889	
		Hohhot segment	E1: 18,750 ± 750 E2: 16,970 ± 960 E3: 14,650 ± 670 E4: 11,820 ± 690 E5: 9450 ± 260 E6: 6830 ± 260 E7: 4500 ± 230	6	0.7495 0.9768 1.1916 0.9979 1.1032 0.9811	
f8	Wutaishan northern piedmont fault	Eastern segment	E1: 6500 E2: 4000 E3: 1480	2	0.9960 1.0040	[23]
f9	Taibai–Weishan northern piedmont fault	Central segment	E1: 7230 E2: 3600 E3: 390	2	1.0614 0.9386	[18]
f10	Yangyuan Basin southern margin fault	Segment A	E1: 20,220 E2: 15,680 E3: 14,247 E4: 8565 E5: 3800	4	1.1060 0.3491 1.3842 1.1608	[18]
		Segment B	E1: 15,845 E2: 10,950 E3: 8970 E4: 6650	3	1.5971 0.6460 0.7569	
f11	Yanggao–Tianzhen northern margin fault	Western segment	E1: 9365 E2: 8760 E3: 7972	2	0.8686 1.1314	[18]
		Eastern segment	E1: 14,512 E2: 12,520 E3: 10,405 E4: 6007	3	0.7026 0.7460 1.5513	
f12	Xuanhua Basin southern margin fault		E1: 8540 E2: 7080 E3: 5310	2	0.9040 1.0960	[24]
f13	Huai–Zhuo Basin northern margin fault	Northern segment	E1: 20,500 ± 1180 E2: 14,500 ± 710 E3: 6700 ± 600 E4: <1310	3	0.9068 1.1788 0.9144	[23]
		Southern segment	E1: 18,750 ± 1400 E2: 16,970 ± 700 E3: 14,650 ± 2100 E4: 11,820 E5: 9450 ± 400 E6: 6830 ±500 E7: <2865	5	0.7466 0.9732 1.1871 0.9941 1.0990	
f14	Xinbaoan–Shacheng fault		E1: 23,205 ± 905 E2: 16,025 ± 615 E3: 8159 ± 500 E4: <7619 ± 95	2	0.9544 1.0456	[24]
f15	Yanfan Basin northern margin fault	Langshan–Fangjiachong segment	E1: 19,850 ± 750 E2: 10,505 ± 497 E3: 4900 ± 400	2	1.2502 0.7498	[24]
		Fangjiachong–Hanhaozhuang segment	E1: 19,850 ± 750 E2: 16,000 ± 1300 E3: 10,505 ± 497 E4: 6599 ± 155 E5: 541	4	0.7976 1.1383 0.8092 1.2550	
		Hanhaozhuang–Xinzhuangpu segment	E1: 19,850 ± 750 E2: 13,600 ± 1000 E3: 6599 ± 155	2	0.9433 1.0567	
		Yanwanggou–Shuigou segment	E1: 32,050 ± 1050 E2: 21,400 ± 900 E3: 10,500 ± 300	2	0.9884 1.0116	
		Langshan–Sangying segment	E1: 27,640 ± 680 E2: 13,950 ± 550 E3: 6850 ± 650 E4: <3663 ± 165	2	1.3170 0.6830	

**Table 3:** Iterative optimization process for statistical determination of coefficient of variation based on $\frac{T}{T_{ave}}$.

Iteration Step	Initial α_0_	α/α_0_ (2 Interval)	α/α_0_ (3 Interval)	α/α_0_ (4 Interval)	α/α_0_ (5 Interval)	α/α_0_ (6 Interval)	Resulting α
1	0.306	0.664	0.782	0.838	0.871	0.894	0.374
2	0.374	0.647	0.769	0.826	0.862	0.884	0.380
3	0.380	0.646	0.766	0.825	0.859	0.882	0.381
4	0.381	0.644	0.766	0.824	0.858	0.883	0.381

## Data Availability

All data used in this paper came from published sources listed in the references.
